# Echinococcosis and Albendazole: A Case for Suitable Treatment

**DOI:** 10.4269/ajtmh.18-0609

**Published:** 2018-08-20

**Authors:** John Horton

**Affiliations:** Tropical Projects, Hitchin, United Kingdom

Although albendazole has been available for human use since 1982 and used for echinococcosis since the mid-1980s, for the most part, it is only approved for the treatment of intestinal helminth diseases as a single dose. Thus, in most countries, the only available presentation is a single 400-mg tablet or two 200-mg tablets. Albendazole is, however, a recommended treatment for several parasitic diseases for which multiple doses are required, and thus, it is often necessary to provide medication in a nonstandard manner. Furthermore, where a multiple dose presentation is available, the approved labeling does not align with the currently recommended long-term regimens. This poses significant challenges for those wishing to treat their patients for echinococcosis.

Manciulli and others,^1^ in this issue, show that even when albendazole is theoretically available, as in Italy, it is not always possible to obtain it, and patients face either intermittent availability or must go to extraordinary lengths to obtain their drug supplies. Irregular treatment is far from ideal in terms of efficacy, and it is likely that some patients suffer reduced efficacy or require more prolonged treatment as a result. This problem is not an isolated one, as it has been reported that similar issues have been faced in many countries where echinococcosis is encountered. Patients and their doctors are dependent on the manufacturers making supplies available in small quantities at irregular intervals, which is difficult logistically and problematic economically. If supplies are not immediately available locally, the only recourse is for the hospital or patients to search outside of their country, often depending today on Internet searches. By contrast, countries such as Argentina and China, with a larger burden of cases, have taken measures to ensure that a supply is available, often using locally produced material.

The previous scenario relates largely to developed countries, where there is good health-care infrastructure and multiple dose packages are potentially available. In many developing countries, however, multiple dose packages are not registered, and patients must use single tablet packs, which are costly when purchased on the open market and are not indicated for the treatment of echinococcosis or other systemic helminth diseases. Albendazole in this form is widely available both from the original producer and from multiple generic companies. However, although generic drugs may be cheaper than the original product, there is no guarantee that they are of sufficient quality to be effective. Gallia et al.^2^ conducted an analysis of a random sample of generic albendazole tablets and found that although some were similar to the original product in dissolution and disintegration tests, others failed badly. Although this study was carried out some years ago, there is no indication that the situation has changed, whereas the number of generic products has increased considerably. One more recent study compared original albendazole with two local generics in Nepal, for both pharmaceutical characteristics and efficacy for soil-transmitted helminth infections, and found that the two generics were inferior,^3^ whereas another study in Ethiopia compared two generics and identified differences in efficacy to be due to differences in dissolution and disintegration of the tablets.^4^ Although it is possible to screen generic products for their pharmaceutical properties using clearly defined methods, such as those defined in the U.S. Pharmacopeia, there is no simple way to evaluate efficacy, other than field testing. Although this might be possible for soil-transmitted helminth infections, the same is not true for echinococcosis. Even providing rational treatment advice based on evidence has proven problematic and taken many years. Therefore, the quality and origin of albendazole must be considered as a surrogate for efficacy when establishing alternative sources of supply.

Even when supplies are assured, there remains the question of the approved indication, which has yet to be resolved. Multidose packs in Europe and elsewhere still carry the advice that treatment should be given in 28-day cycles for a maximum of three cycles, a recommendation that dates back to the 1980s, when albendazole was first registered for the treatment of echinococcosis. For the last 25 years, advice has been to use albendazole continuously for longer periods, with monitoring to pick up any toxicity. Following this advice is technically off-label, and in some countries, this precludes reimbursement for drug costs. There is, therefore, an urgent need to update prescribing information on multidose packs so that patients receive the most appropriate treatment and to ensure that treatment-appropriate packaging is available in endemic countries.

Finding solutions to the problem of limited albendazole availability for long-term dosing does not seem to be amenable to traditional public health approaches. The technical manual^5^ produced by Wold Health Organization provides best advice, but exists outside drug regulatory rules. This manual is due to be updated in 2018.^6^ As albendazole is now almost 40 years old, there is limited desire to spend time and money revising the prescribing information, especially in face of the large number of existing generics available, all of which would have to change as well, country by country. It will, therefore, be necessary to ensure that all practitioners and treatment centers are aware of current recommendations and to encourage local authorities to accept and follow these recommendations. Ensuring that adequate, appropriate, and cost-effective supplies of albendazole are available will remain a challenge for those treating and those suffering from echinococcosis, and producers must be encouraged to provide appropriate dosing regimens. Without this, echinococcosis will remain a much neglected condition.
